# Polymerization mechanism of the *Candida albicans* virulence factor candidalysin

**DOI:** 10.1016/j.jbc.2024.107370

**Published:** 2024-05-13

**Authors:** Katherine G. Schaefer, Charles M. Russell, Robert J. Pyron, Elizabeth A. Conley, Francisco N. Barrera, Gavin M. King

**Affiliations:** 1 Department of Physics and Astronomy, University of Missouri, Columbia, Missouri; 2 Department of Biochemistry & Cellular and Molecular Biology, University of Tennessee, Knoxville, Tennessee; 3 Genome Science and Technology, University of Tennessee, Knoxville, Tennessee; 4 Department of Biochemistry, University of Missouri, Columbia, Missouri

**Keywords:** yeast, single molecule biophysics, cyclization, branching, looping, debye length, ionic strength

## Abstract

*Candida albicans* is a commensal fungus that can cause epithelial infections and life-threatening invasive candidiasis. The fungus secretes candidalysin (CL), a peptide that causes cell damage and immune activation by permeation of epithelial membranes. The mechanism of CL action involves strong peptide assembly into polymers in solution. The free ends of linear CL polymers can join, forming loops that become pores upon binding to membranes. CL polymers constitute a therapeutic target for candidiasis, but little is known about CL self-assembly in solution. Here, we examine the assembly mechanism of CL in the absence of membranes using complementary biophysical tools, including a new fluorescence polymerization assay, mass photometry, and atomic force microscopy. We observed that CL assembly is slow, as tracked with the fluorescent marker C-laurdan. Single-molecule methods showed that CL polymerization involves a convolution of four processes. Self-assembly begins with the formation of a basic subunit, thought to be a CL octamer that is the polymer seed. Polymerization proceeds *via* the addition of octamers, and as polymers grow they can curve and form loops. Alternatively, secondary polymerization can occur and cause branching. Interplay between the different rates determines the distribution of CL particle types, indicating a kinetic control mechanism. This work elucidates key physical attributes underlying CL self-assembly which may eventually evoke pharmaceutical development.

The fungus *Candida albicans* is responsible for 150 million mucosal infections and ∼200,000 deaths annually (1, 2). Although *C. albicans* typically resides as a harmless commensal, candidiasis can occur in the form of a superficial mucosal infection such as oral and vaginal candidiasis (3) or as deadly invasive candidiasis, which has a 50% mortality rate (4). *C. albicans* harms host cells by releasing candidalysin (CL), a peptide that is a key virulence factor for the infection that forms membrane-damaging pores in epithelial membranes (5, 6, 7, 8). The mechanism by which CL damages cell membranes was recently characterized and found to be novel in its mode of action (9). Unlike most pore-forming peptides and proteins, CL self-assembly does not require membranes; most membrane-damaging peptides require membrane binding to initiate assembly (10), while CL spontaneously forms higher-order complexes in solution (9). CL oligomers are prevalent and spontaneously assemble into long polymers that eventually close to form loops, which are thought to become pores once they localize to the membrane. Most antifungals currently available are non-specific and harm the commensal mycobiome, causing side effects (11). Developing a treatment that targets a membrane-damaging virulence factor is likely to offer therapeutic advantages. We reasoned that understanding the mode of action of CL can lead to the development of an effective *C. albicans* therapy consisting of blocking the assembly of this virulence factor before it causes membrane damage.

We sought to understand how CL self-assembles prior to interacting with the membrane. We employed an array of biophysical tools and high-resolution molecular imaging techniques to quantify the oligomeric intermediates and polymers that CL forms over time and characterize key kinetic parameters. Studying peptide oligomerization provides its own set of obstacles (12). Though we understand the self-assembly processes of well-characterized biological filaments like actin (13) or the amyloid family of peptides (14), we had to employ innovative methods due to the complexity of CL polymerization. Our combination of high-resolution, single-molecule techniques paired with sensitive bulk-solution experiments allowed us to identify specific steps in the CL self-assembly process. Here we show that CL self-assembly is under kinetic control and describe the timescale in which this process occurs and deduce characteristic kinetic rates.

## Results

### CL polymerization is under kinetic control

**Figure 1:**
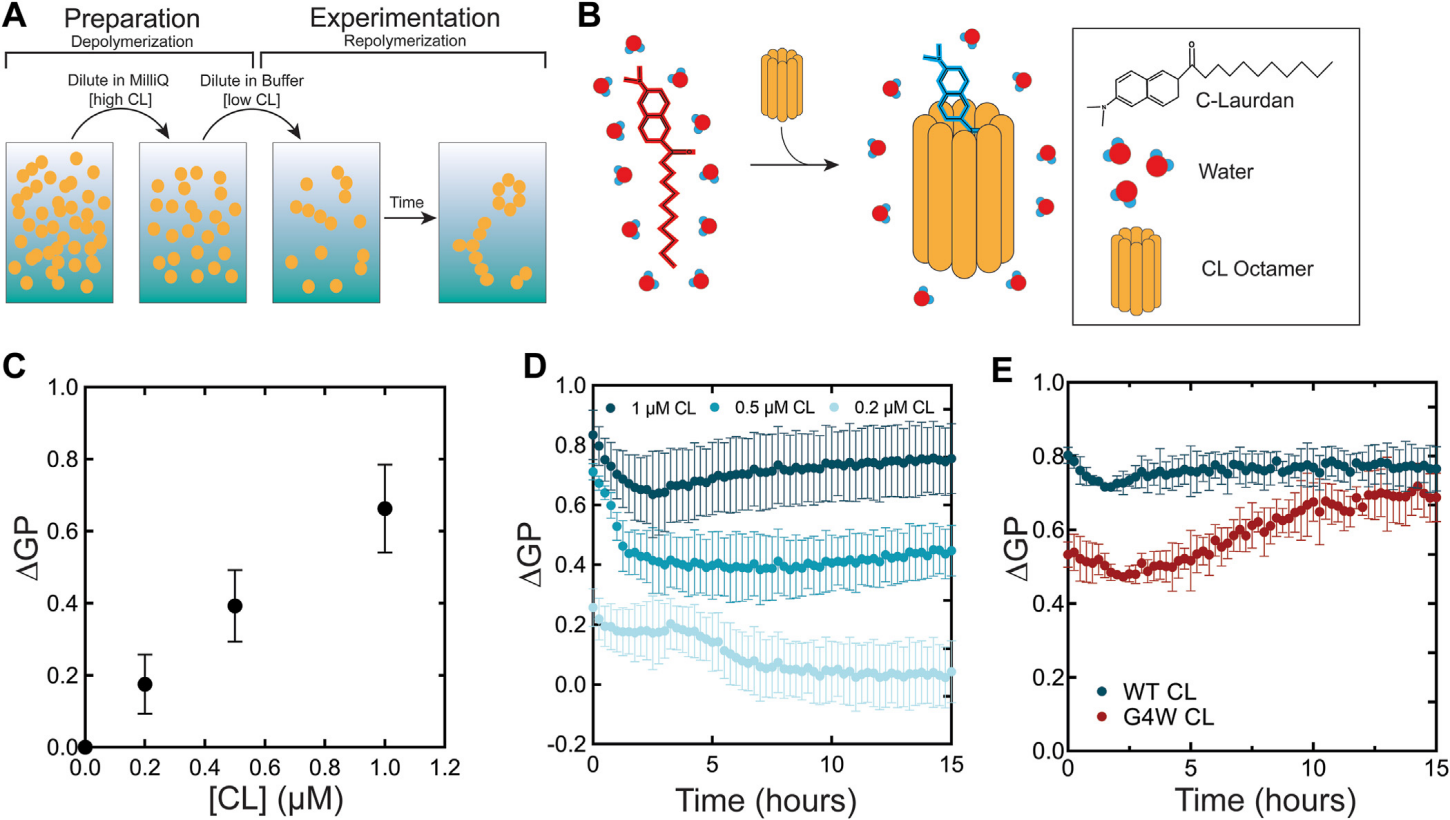
**C-laurdan reports on CL polymerization.***A*, sample preparation strategy to study CL kinetics. Lyophilized CL is resuspended in MilliQ water and diluted to 100 μM for storage. CL is diluted further in MilliQ before being introduced to the working buffer at the desired concentration. *B*, a hypothetical model depicting the fatty acid chain of the polarimetric dye C-laurdan interacting with the hydrophobic moiety of a CL octamer. This interaction induces a partial desolvation of the dye that causes an increase in ΔGP, which is a measure of C-Laurdan spectral emission shift, as described in Experimental procedures. *C*, ΔGP-values increase at higher [CL]. Data points were recorded after a 4-h incubation of CL with 1 *μ*M C-laurdan. *D*, C-laurdan tracks CL polymerization kinetics. ΔGP-values reflect dynamic changes in C-laurdan solvation status over time. *E*, polymerization-deficient mutant G4W undergoes hindered ΔGP increase compared to WT CL at 1.0 uM peptide concentration. Error bars represent the standard deviation of three independent experiments.

We used the fluorescent dye C-laurdan to track CL polymerization in solution. C-laurdan is typically used for discerning lipid packing information in membranes, as it reports on the hydration of its local lipid environment – characterized by a 50 nm blue-shift when it is sequestered from water (15, 16, 17). The dual-emission fluorescence of C-laurdan is typically converted into a generalized polarization (GP) value that transforms spectral information on a linear scale (18, 19). As such, GP reports on the degree of interactions between the dye and water: a GP value of −1 corresponds to full water exposure, while a higher GP reflects a less polar environment. We prepared CL samples in buffer from concentrated stocks (Fig. 1*A*), and to our surprise we observed that CL interacts strongly with C-laurdan in the absence of a membrane, as indicated by a large shift in the fluorescence emission spectra (Fig. S1) and the resulting GP increase (Fig. 1*C*). This interaction is likely mediated by the dye’s binding to a yet-undetermined hydrophobic pocket on CL, leading to a partial dehydration of the CL-bound dye (Fig. 1*B*).

With this finding, we posited that C-laurdan could be useful in studying CL polymerization. When we increased the concentration of CL to promote polymerization (9), we observed an increase in GP, suggesting that greater sequestration of the dye from water occurs upon CL polymerization (Fig. 1*C*) due to the increase in large CL polymeric assemblies around the dye. We calculated the change in GP value of our samples compared to control samples that only contained C-laurdan (see Experimental procedures). The use of the resulting ΔGP parameter should help to avoid confusion with the standard GP parameter used in C-laurdan membrane studies.

We performed a long time-course ΔGP experiment to study the dynamics of CL self-assembly (Fig. 1*D*). There were distinctive differences in the shapes of the ΔGP curves at a range of CL concentrations, indicating changes in the kinetics of CL polymerization. CL at 0.2 μM underwent a slow ΔGP decrease that stabilized after about 10 h (Fig. 1*D*). Results were different at higher concentrations, where the ΔGP was higher and recovered after an initial decrease. We attribute the ΔGP decrease phase to polymer disassembly, possibly of preformed polymers in the initial concentrated stock, but note that it is difficult to rule out the possibility of dye-related artifacts. At higher CL concentrations this phase reverses over time.

We also incubated C-laurdan with the G4W CL variant, which is deficient in self-assembly and membrane damage (9). The ΔGP data indicates that G4W experiences slower polymerization (Fig. 1*E*), consistent with expectations (9). However, our data reveal that G4W is not impaired for polymerization, although this process is quite slow. Under our experimental conditions G4W does appear to form polymers after an extended lag phase (∼4 h). Overall, the data indicate that C-laurdan can be used to track CL polymerization in solution. Though the fluorescence polymerization assay cannot resolve what specific structures CL forms, the results indicate that long timescale processes are at play. We next sought to understand the kinetic underpinnings of CL self-assembly in solution using higher resolution techniques.

**Figure 2:**
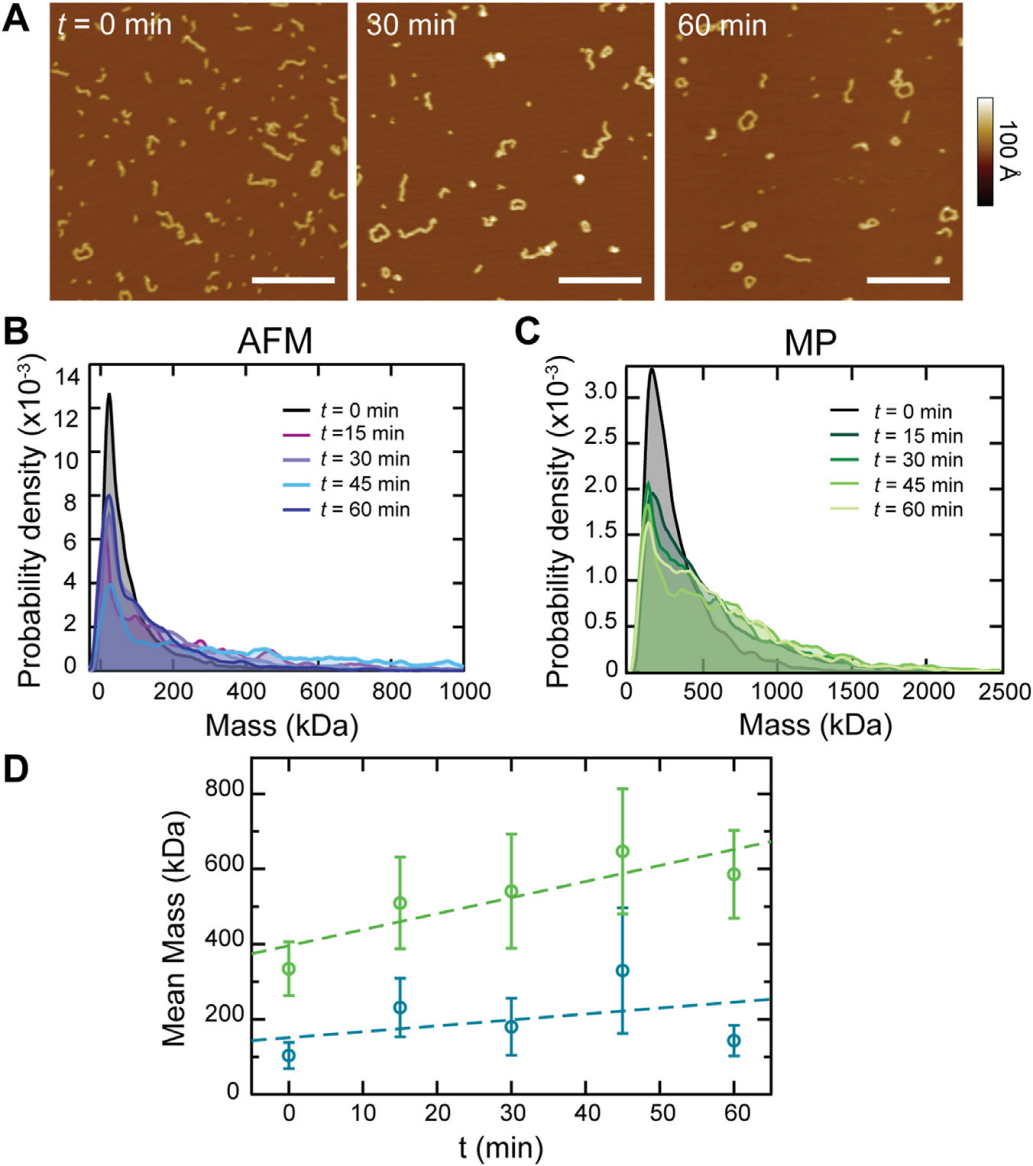
**Time dependence of CL polymerization.***A*, AFM images demonstrate progressive polymerization of CL subunits over time; scale bars = 200 nm. *B*, histograms of AFM particle mass, calculated from volume data, show progressive shifts to higher mass over time. *C*, MP data show a similar progression. *D*, the mean mass and standard deviation from MP (*green*) and AFM (*blue*) data were calculated at different time points. *Dashed lines* overlaid to guide the eye; [CL] was 330 nM.

To gain a deeper understanding of CL polymerization, we employed two single-molecule techniques established to be effective for studying this system—atomic force microscopy (AFM) and mass photometry (MP). By performing AFM imaging of 330 nM CL after various incubation times after dilution (*t* = {0, 15, 30, 45, 60} min), the progression of polymerization was visualized (Fig. 2*A*). Without incubation (t = 0 min), short 1 to 4 subunit polymers dominate each image, accompanied by a few longer polymer and loops, which are pore-competent species (9). Images taken at longer incubation times display particles of larger size and an increased number of loops. In previous work, we identified successive peaks in a volume histogram (*t* = 0 min) corresponding to the addition of a fundamental subunit, which our data indicate is a CL octamer (9). Particle mass can be estimated from AFM data (20). By fitting a line to the first four peak positions, we determined a conversion factor from volume to number of subunits, 274 ± 8 nm^3^ per subunit (Fig. S2). Using this conversion, we calculated the number of subunits in each AFM feature and converted to mass assuming that each subunit is an octamer (the molecular weight of an individual CL peptide is 3310 Da). The resulting histograms of particle mass for each time point (Fig. 2*B*) display a main peak centered around 22 kDa, in approximate agreement with the expected octamer, and a long tail reaching hundreds of kDa. Over time we observed a reduction in the intensity of the primary peak and growth of the tail.

Next, we performed MP experiments under identical conditions (Fig. 2*C*). MP also determines the mass of the particles, although this instrument is only able to resolve particles larger than 40 to 50 kDa (14), and therefore it will not detect the CL octamer. However, MP offers excellent resolution to identify large particles. MP results generally agreed with the AFM data. We observed that the main MP peak at t = 0 was broad and appeared to be a convolution of several populations that are too closely spaced to be resolved. At later times a sharper peak at low mass was resolved when some populations shifted towards higher masses. Comparing the average mass calculated from AFM features and MP (Fig. 2*D*), the latter reported a higher average particle mass, as expected. However, the two techniques agreed on identifying pre-existing polymers before incubation starts which suggests that the 100 μM CL aliquots we employ to initiate experiments contain a significant amount of CL polymers (Fig. 1*A*). This observation supports the initial disassembly phase observed in the fluorescence data (Fig. 1*E*).

### Single-molecule study of CL polymerization

While tracking CL self-assembly over time with C-laurdan, we observed that each condition had a baseline ΔGP and trajectory that depended on its concentration (Fig. 1*D*). The two higher CL concentrations (0.5 μM and 1 μM) exhibited trajectories indicative of slow CL polymer growth. In contrast, the ΔGP data for the lowest CL concentration (0.2 μM) was indicative of disassembly or depolymerization. To initiate polymer growth, it appears that CL must exceed a minimum concentration.

**Figure 3:**
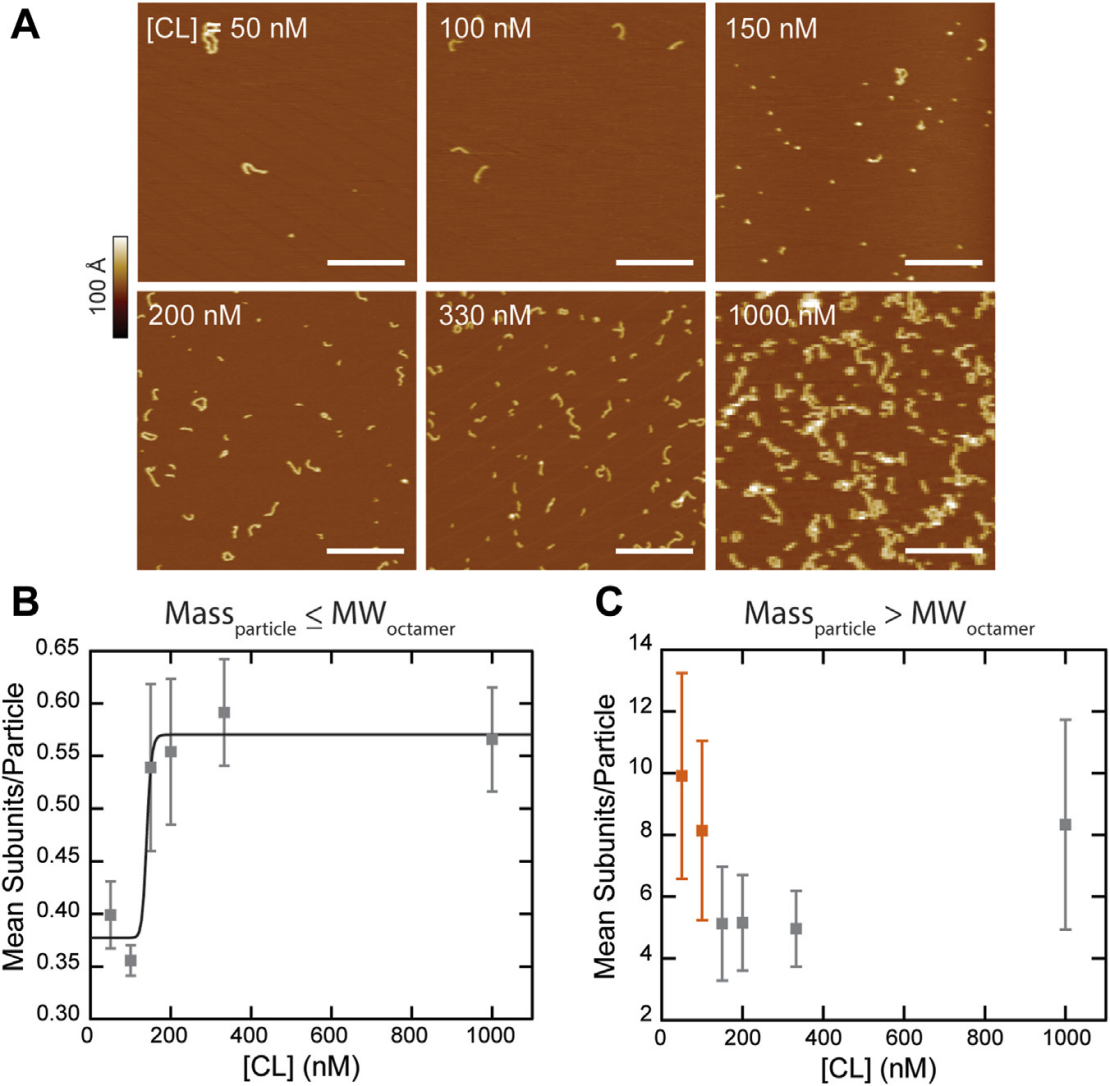
**Concentration effects on CL polymerization.***A*, AFM images show CL particle behavior at increasing concentrations; scale bars = 200 nm. To distinguish between subunit formation and large-scale polymerization, particle distributions are filtered and average subunits/particle plotted for particles with mass (*B*) less than or equal to an octamer (*open squares*) and (*C*) greater than the octamer (*closed squares*). The particles less than or equal to an octamer (*B*) exhibit a sigmoidal trend that was fitted with a Hill equation. At concentrations less than 150 nM, very few particles are observed per area and pre-formed complexes dominate the average mass of particles larger than an octamer (*C*, *orange squares*). All error bars are standard deviations.

To identify the structures associated with the ΔGP kinetics assay and MP data, AFM was performed at a variety of CL concentrations with no pre-incubation ([CL] = {50, 100, 150, 200, 330, 1000} nM). Figure 3*A* shows an increase in the number and size of CL particles as the concentration increases. To quantify how particle size changed with concentration, the mean number of subunits per particle was calculated from the particle volume using the same conversion factor as before (274 ± 8 nm^3^ per subunit). We further differentiated between two processes: octamer formation and polymerization (*i.e.*, binding of two or more octamers). To do so, we sorted particles into two groups: those that had sizes less than or equal to an octamer (Fig. 3*B*) and those that had sizes greater than an octamer (Fig. 3*C*; see Fig. S3 for all features combined).

In the case of particles smaller than or equal to an octamer, the curve displayed sigmoidal behavior. Fitting with a Hill equation yields the concentration of CL at half the maximum subunit per particle, [CL]_*half*_ ≈ 140 nM. It appears that at CL concentrations below this threshold, the majority of CL is not yet involved in the fundamental nucleation particle required for polymerization, which we take to be an octameric subunit throughout this work. Once this particle is formed, then CL polymerization proceeds as expected for larger complexes (Fig. 3*C*, grey). Notably, for concentrations less than 140 nM, large particles seem to dominate the average (Fig. 3*C*, orange), though the overall number of these particles observed is quite low. We hypothesize that these complexes, which are also likely to be present in higher concentration preparations, were formed during the initial CL hydration/freezing/dilution steps (Fig. 1*A*) and are further evidence of a difficult-to-control *t* = 0 point. Concentrations higher than 140 nM showed the expected behavior of an increase in particle size as more octamers are made available. Overall, our results reveal a complex dynamic interplay between CL polymeric species.

### CL polymerization is modulated by electrostatics

**Figure 4:**
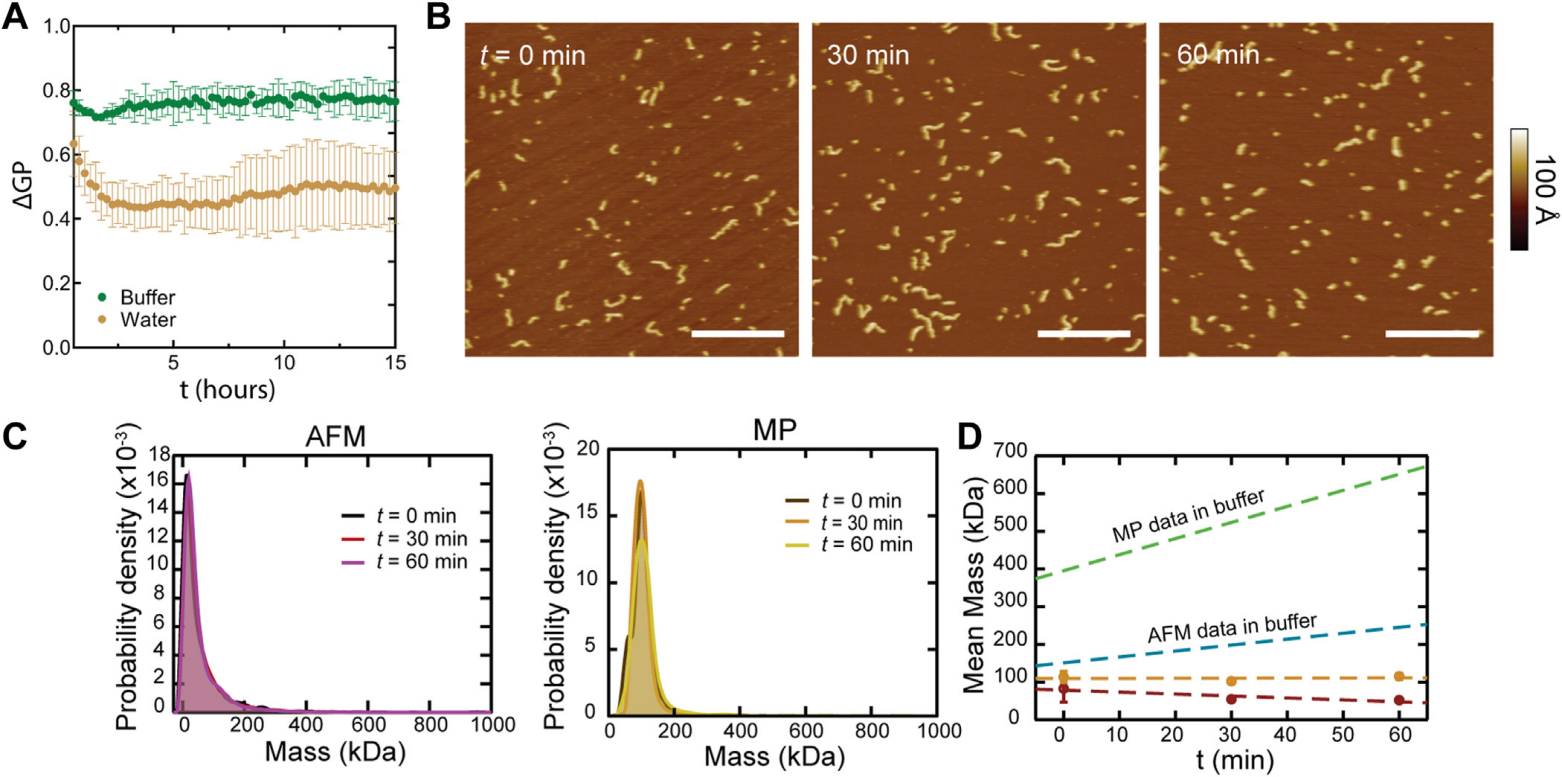
**Low salt conditions reduce polymerization.***A*, ΔGP kinetic assay of CL (1 μM) self-assembly measured in the presence (*green*) and absence (*gold*) of salt. Error bars are the standard deviation for three biological replicates. *B*, CL diluted and incubated in ultrapure water for 0 and 60 min were imaged *via* AFM, revealing low polymerization; scale bars = 200 nm. *C*, AFM images are analyzed and mass histograms show a narrow peak with a minor shoulder. Similar results were observed in MP, illustrated by histograms in which there is no appreciable increase in larger mass features over time. *D*, the average mass determined from MP (*gold*) and AFM (*red*) is plotted over time, showing little to no change when compared to data collected in buffer (*green* and *blue dashed lines*).

To investigate the nature of the non-covalent interactions formed upon CL polymerization, we tested the effect of ionic strength. When we performed the ΔGP assay in MilliQ water, we observed lower values compared to buffer conditions (Figs. 4 and S4). Additionally, the disassembly step was pronounced. These results indicate that CL forms smaller polymeric assemblies in water compared to buffer, revealing that modulation of ionic conditions affects CL assembly.

To determine the extent to which electrostatic interactions play a role in the polymerization process, CL feature size was assessed *via* AFM and MP in ultrapure water after different periods of incubation (Fig. 4*C*). In contrast to results obtained in buffer, CL features did not grow over time in water. AFM images showed mostly short, linear polymers and very few loops (Fig. 4*B*). Histograms of mass calculated from AFM and MP data (Fig. 4*C*) all have a sharp, primary peak centered around 16 kDa for AFM and 99 kDa for MP. When the average mass for each method is plotted over time, no growth is detected *via* either technique (Fig. 4*D*). Due to acidity of ultrapure water (pH ∼5), we also performed experiments in pH five buffer to test whether acidity itself affects CL polymerization due to protonation of the C-terminal group. While pH had a slight dampening effect on subunit (octamer) formation, polymeric assembly of the subunits over time remained similar to buffer at physiological pH (Fig. S5), indicating that acidity does not play a major role in polymerization.

The bending stiffness of charged polymers is known to vary inversely with ionic strength (21, 22). To evaluate this effect for CL, polymer backbone traces extracted from AFM data were used to calculate the persistence length, *l*_p_. The analysis (Fig. S6) revealed a ∼40% enhancement in bending stiffness (*l*_p_) in ultrapure water. Altogether, the results indicate that ionic strength (150 mM NaCl in this work) is a defining factor for CL polymers. Furthermore, since high ionic strength potentiates the hydrophobic effect, the results suggest that hydrophobic interactions are required for CL polymer growth.

### Rates of formation for different particle types

**Figure 5:**
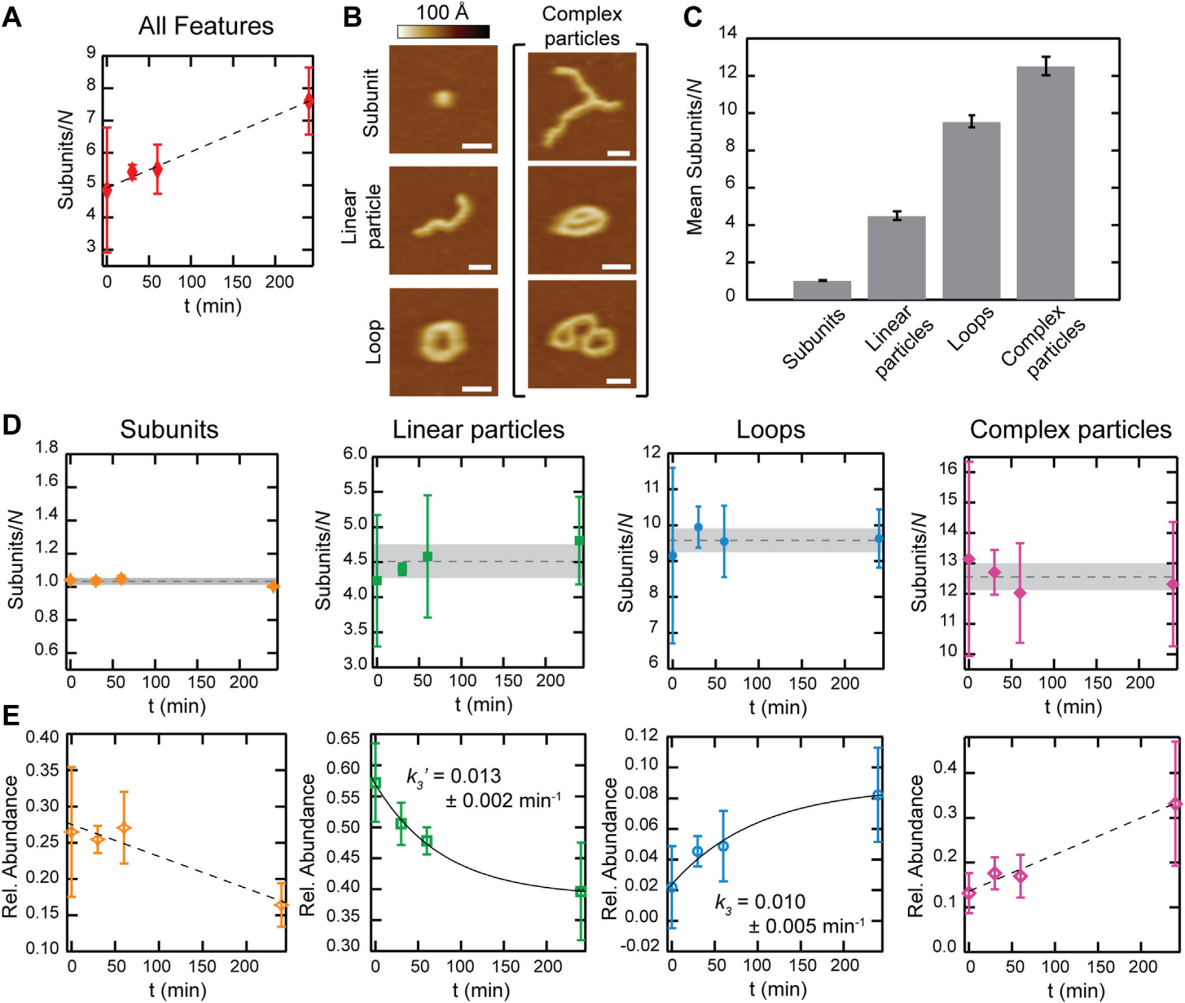
**Kinetics of particle types.***A*, the average subunits per particle for all features (*N* = 9623) was quantified and plotted over time at 330 nM CL. A linear fit (*dashed line*) guides the eye. *B*, representative images of identified particle types are shown, with the simplest being the subunit, linear particle, and loop (*left*). Complex particles are defined as having one or more branch points and have a variety of morphologies, a selection of which is displayed (*right*); scale bars = 20 nm. *C*, mean subunit/*N* for particle type. Error bars are the standard deviation. *D*, plots of subunit/*N* as a function of time exhibit minimal fluctuations about the mean for each particle type. *Horizontal dashed lines* and *shaded boxes* indicate the mean and standard deviation, respectively. *E*, the abundance of each particle type relative to all features is displayed *versus* time. *Dashed lines* guide the eye on the plots of the subunits and complex particles; since the abundance of linear particles and loops plateaus, they were fitted with exponential functions (*solid lines*). Effective rate constants extracted from the fits are displayed. Error bars are standard deviation.

An advantage of visualization of CL particles in AFM is the ability to sort features into categories and evaluate their kinetic rates of formation. We used a custom algorithm to sort CL particles and determine salient physical quantities including the shape and contour lengths of each. Particles were sorted into four types: basic subunits (octamers), linear particles, loops, and complex particles (Fig. 5*B*). The complex particle type is a broad category defined as any particle that exhibits branching (presumably through a secondary polymerization interface), multiple loops, or a combination of these. For this analysis, no distinction was made between secondary polymerization and overlapping particles.

To examine particle size over time, we quantified the average number of subunits per particle (subunits/*N*). The number of subunits in a particle was determined from the contour length using simulated AFM images of the octamer/polymer structures (9). Due to tip convolution, this value depends on both the particle morphology and the tip shape and was approached differently depending on the particle type (See Fig. S7). As expected from our analysis of mass over time (Fig. 2), the average subunits/*N* increase over time for all features (Fig. 5*A*). When deconvolved by shape, new trends emerge. Unsurprisingly, no increase in length is observed for the basic subunit. Similarly, there was no discernible trend in particle size for the rest of the particle types (Fig. 5*D*). Therefore, we determined a time average of the number of subunits for each particle type (Fig. 5*C*): 1.03 ± 0.02 for octamers, 4.5 ± 0.3 for linear polymers, 9.6 ± 0.3 for loops, and 12.5 ± 0.5 for complex particles.

Since in our experimental conditions, we do not observe growth in the length of the polymers, these results suggest that over time there is an interconversion between particle types. To evaluate this idea, we determined the relative abundance of each particle at all time points. We observed that individual subunits decreased from 27% to 16% (Fig. 5*E*). Linear particles also experienced a decrease in abundance over time. In contrast, the number of both loops and complex particles increased. Loops increased from 2% to 8%, which is still a low overall abundance. This is the case because most loops are involved in higher order complexes (*i.e.*, complex particle types), which grow in number significantly over time. Based on these quantifications, the increase in average particle size for all features over time (Figs. 2*E* and 5*A*) can be attributed to an increase in the overall number of larger particle types at the expense of smaller particles.

## Discussion

We investigated the kinetics of CL self-assembly *via* a new C-laurdan fluorescence polymerization assay, mass photometry, and atomic force microscopy. Our experiments revealed a detailed picture of CL behavior in solution. C-laurdan is a fluorescent probe that we serendipitously identified to interact with CL; that interaction was useful in determination of polymerization levels and kinetics in solution (18, 23, 24). This is a new application of this dye, which is often employed to report on membrane properties, such as fluidity and lipid packing. We suggest that caution should be exercised when using C-laurdan to study membrane properties in samples that contain amphipathic or hydrophobic polypeptides or polymers, since C-laurdan might additionally report on non-lipid environments. Our ΔGP data additionally provide a consistency check for mass photometry and AFM, since both of these single molecule approaches require a proximal surface. We observed positive shifts in ΔGP when C-laurdan was incubated in the presence of CL, indicating higher sequestering of the dye from water, which points to CL assemblies interacting with the dye. This conclusion was supported by measurements with a less toxic mutant G4W, known to have diminished self-assembly potential (9). The long incubation period over which CL growth was observed demonstrates a slow self-assembly rate.

Direct visualization of CL polymerization complemented the fluorescence assay and showed an increase in larger features over time. The analysis led us to identify four underlying reactions: octamerization (*k*_1_), primary polymerization (in which octamers are added, likely in an end-to-end orientation, *k*_2_), cyclization (*k*_3_), and secondary polymerization (branching, *k*_4_). Single-particle AFM imaging allowed us to categorize particles into specific types, which are the products associated with each of the processes: subunits, linear particles, loops, and complex particles (Fig. 5). Surprisingly, quantification of the size evolution of each particle type over 4 h showed generally stable particle sizes for the linear polymers, loops, and branches. However, upon examining the relative abundance of each, we found that the distribution of particle types changed over time. Octamer subunits were progressively used up in the formation of higher order particles. The linear particles became less abundant, whereas the number of loops and complex particles increased. Although no determination can be made as to the rates of octamerization or primary polymerization, *k*_1_ or *k*_2_, the high abundance of octamers and linear polymers at *t* = 0 min indicates that these reactions proceed on a faster timescale than the events leading to loop and complex particle formation. We posit octamerization to be the fastest event as subunits are required for larger assemblies (Fig. 3).

We observed a plateau in the abundance of loops, which indicates that the apparent cyclization rate constant, *k*_3_, is within the timescale of measurement. We emphasize the importance of *k*_3_ in the underlying CL mechanism during *C. albicans* infection. This cyclization rate defines the CL topological transition timescale from linear polymer to closed loop; it is these loops that are thought to go on to become host cell membrane-damaging pores (9). The precise value of *k*_*3*_ is difficult to extract from our measurements, for as loops are formed, they are also being used up in secondary polymerization events that create complex particles. However, we can make some assumptions about cyclization. This is a first-order reaction, as it involves a single linear polymer forming a loop, making it exponential in time. Fitting the loop abundance data with an exponential rise to maximum (Fig. 5*E*) yields an apparent *k*_*3*_ = 0.010 ± 0.005 min^−1^ (in 10 mM Hepes, 150 mM NaCl, pH 7.3, at a CL concentration of 330 nM). If we assume that branching occurs with similar probabilities for both linear particles and loops, then the loss should create an equal offset in the two particle types. We therefore compared the apparent cyclization rate to the rate of linear particle decay, *k*_*3*_^*’*^ = 0.013 ± 0.002 min^−1^, and found that the two values agree within uncertainty, reflecting a “forward” rate of cyclization or a successful conversion of linear to looped polymers. It is important to note that *k*_*3*_ is highly dependent on solution conditions and approaches zero when CL buffer is replaced by ultrapure water. For additional context, the upper limit of end-to-end cyclization based on a diffusion process can be estimated as *k*_cy_ ≈ D/<*R*^2^>, where *D* is the polymer translational diffusion coefficient and <*R*^2^> is the mean square end-to-end distance (25, 26). Plugging in approximate values (see Experimental procedures) yields a theoretical diffusion limited *k*_cy_ that is seven orders of magnitude faster than the measured *k*_3_. We conclude that there are significant factors thwarting the CL cyclization pathway under our experimental conditions.

**Figure 6:**
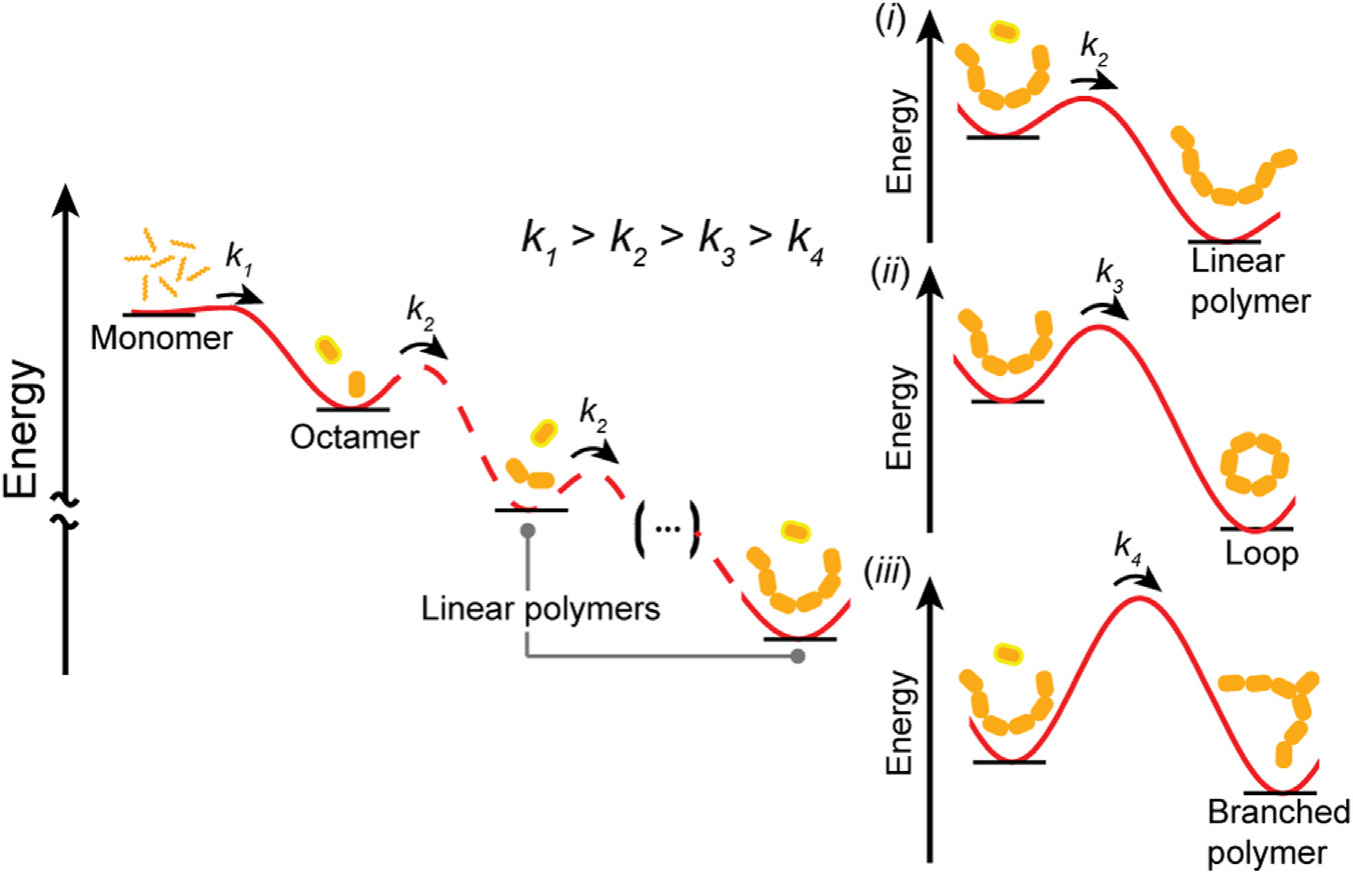
**Model of multiphasic CL polymer assembly.** An energetic diagram describes the proposed model for the assembly of CL polymers starting with the CL monomer with highest energy and fastest assembly into an octamer (k_1_). Octamers may then join to form a polymer of *n* subunits, displayed here with *n* = 2 and 6. The rate of this type of particle formation is labeled *k*_2_. From a linear polymer, three possibilities exist: (*i*) continued polymerization at either free end with rate *k*_2_, (*ii*) closure of free ends to form a more energetically stable loop with rate *k*_3_, or (*iii*) addition of an octamer at a secondary site, forming a branched polymer. The energy barrier for this event is expected to be higher, thus resulting in a slow *k*_4_.

The rate of secondary polymerization, *k*_*4*_, cannot be easily determined, as the category of complex particles is broad and contains particles with several branching events. Since there is no plateau for the formation of complex particles in the period observed, this process appears to have the slowest rate. Hence, in bulk polymerization assays, branching would appear as the rate-limiting step. A summary of these conclusions is presented in a schematic energy diagram (Fig. 6). Energy barrier heights correspond to the hypothesized relative rates. Energy levels for each successive state are assumed to be lower than the previous, as the reaction proceeds spontaneously in physiological buffer.

In addition to kinetic information, the nature of several steps in the CL polymerization process was also revealed. The driving force behind octamer-octamer interactions was elucidated by experiments conducted in ultrapure water (Fig. 4). By reducing the concentration of free ions in solution during CL incubation, primary polymerization was greatly reduced. Evidently CL polymer growth is stunted when long-range electrostatic interactions are not well-shielded, due to the large ∼1 μm Debye length of ultrapure water (27). Another observation was a slight reduction in octamerization as evinced by the shift in location (to about 16 kDa) of the primary peak in the AFM mass histogram for ultrapure water experiments (Fig. 4*C*), possibly indicative of a 5-mer. This is substantially smaller than the primary peak of the AFM mass histogram in buffer, which was located at ∼22 kDa (Fig. 2*B*). The change in seed particle size likely contributes to the suppression of primary polymerization. Additionally, cyclization was almost completely abolished in ultrapure water. This effect comes about from two sources. First the suppression of linear polymer growth (see Fig. S8) leads to fewer polymers with sufficient length to close into a loop. Second, polymers that are sufficiently long are more rigid and hence less likely to bend into a closed-loop configuration, since the persistence length of CL polymers was found to be 40% larger in water (see Fig. S6).

To summarize, using a variety of biophysical techniques, we have probed the kinetics and interactions of CL self-assembly. The dependence of the particle distributions at certain time points on the relative rates of the processes indicates that CL polymerization is under kinetic control. We were surprised to determine the low frequency of isolated pore-competent loops, which represent only 2% to 8% of all CL species. This suggests that in physiological conditions, biological factors likely exist that enhance pore-inducing loop formation and maturation. Additionally, we show that octamerization, primary polymerization, cyclization, and secondary polymerization are all affected by electrostatic interactions to varying degrees. Further work will be required to identify specific hydrophobic interactions which likely stabilize CL polymers. Self-assembly is the first step that CL undergoes in the process of forming pores that cause membrane disruption and immune activation (9). Our data provide a deeper understanding of this process, paving the way for the development of new anti-candida pharmaceuticals that disrupt CL polymerization and its toxic effects.

## Experimental procedures

### Sample preparation

CL peptide was synthesized by Fmoc chemistry (Peptide 2.0), and >95% purity was assessed by HPLC and MALDI-TOF. CL was resuspended in ultrapure water (Milli-Q, 18.2 MΩ∗cm) to form 100 μM stocks, which were aliquoted and stored at −80 °C. At the time of experimentation, a CL aliquot was thawed and diluted in CL buffer (10 mM Hepes, 150 mM NaCl, pH 7.3) to the desired concentration. This step was defined as *t* = 0 min. The solution was then held at room temperature for the allotted incubation time (Fig. 1*A*).

### Mass photometry

Mass Photometry was performed as described previously (9) with the following modifications. CL Milli-Q stocks were diluted to 330 nM in CL buffer at the time of experimentation and measured in 15-min increments for 1 h. All measurements were done in triplicates. Smooth histograms were generated using kernel density estimation with Epanechnikov kernels in Igor Pro seven software.

### Fluorescence polymerization assay

C-laurdan (Tocris, CAT# 7273) was dissolved in chloroform to 1.1025 mM (ε = 12,200 M^−1^ cm^−1^) and further diluted with ethanol to create a 100 μM stock (in a 91% ethanol, 9% chloroform solution). C-laurdan organic solvent stocks were diluted at least 100-fold in aqueous solution. Samples were prepared in CL buffer with 1 μM C-laurdan. The emission spectra from 400 to 600 nm were read on a Cytation five plate reader (BioTek) using an excitation wavelength of 350 nm. Spectral blanks without dye were subtracted. GP values were calculated using the following equation:

(1) $$\text{GP}=\left(I_{\text{blue}}-I_{\text{red}}\right)/\left(I_{\text{blue}}+I_{\text{red}}\right)$$

Where, I_blue_ is the summation of fluorescence intensity (F.I.) values within the emission range of 420 to 460 nm and I_red_ is the summation of F.I. values within the emission range of 470 to 510 nm. ΔGP was calculated by subtracting the GP of control samples containing only C-laurdan from the experimental GP value. In control conditions lacking peptide, we observed a GP increase over the first 2 to 3 h, suggesting evolution in baseline C-laurdan molecular assemblies during this time (Fig. S1). However, this signal stabilizes after 3 h. Further investigation is needed to understand and control this evolution.

### AFM imaging and analysis

90 μl of CL solution were deposited on freshly cleaved mica discs and incubated for an additional 10 min to adhere to the surface. Following incubation on the surface, loosely bound particles were washed away *via* buffer exchange (90 μl of CL buffer exchanged across the sample 5–6 times). For assays performed without salt, CL was diluted and rinsed in ultrapure water instead of buffer. Following previous work (28, 29), all images were acquired in CL buffer using biolever mini tips (Bruker, k ∼ 0.1 N/m, f_o_ ∼ 25 kHz in fluid) on a commercial instrument (Asylum Research Cypher) in tapping mode. Tip-sample forces were kept below 100 pN to reduce the likelihood of protein deformation. To quantify geometric parameters such as particle height and volume, images were processed and analyzed using the built-in software (Asylum Research, Inc). In our analysis of CL particle size *versus* concentration, we fit sigmoidal relationships with a general Hill Equation with the following parameterization:

(2) $$y=y_{\min}\;+\;\frac{y_{\max}-y_{\min}}{1+{\left({\left[\text{CL}\right]}_{half}/\left[\text{CL}\right]\right)}^{n}}$$

Here, *y*_*min*_ and *y*_*max*_ and the minimum and maximum values of the *y* parameter (mean subunits/particle), [CL]_*half*_ is the concentration of CL at halfway between *y*_*min*_ and *y*_*max*_, and *n* is the Hill coefficient. A custom algorithm was implemented in Igor Pro 7 (Wavemetrics) to sort particles, calculate contour lengths, end-to-end distances, and persistence lengths using

(3) $$<R^{2}>=2{sl}_{p}L\left[1\;-\;{sl}_{p}/L\left(1\;-\;\exp\left(\;-\;\frac{L}{sl_{p}}\right)\right)\right]$$

which is derived from the worm-like chain model (30). Here, *s* is a surface parameter which is set equal to two for polymers equilibrated on a 2-dimensional surface (31). Briefly, the algorithm skeletonizes the image, determines end points and branch points, then uses a pathfinding process to determine the contour length of the individual particles. To determine the rates, the results were fitted with an exponential function:

(4) $$y=y_{0}\;+\;Ae^{-kt}$$

Here, *y* and *y*_*0*_ are parameters that represent particle abundance and the corresponding intercept. The amplitude is *A* and the rate is *k*. For calculating the theoretical diffusion-limited rate of cyclization, *k*_cy_, the translational diffusion coefficient of CL polymers was estimated to be *D* ≈ 5 × 10^−8^ cm^2^/s based on results (32) from a model charged polymer with similar molecular weight (500 bp DNA) and the mean square end-to-end distance was calculated *via* Equation 3 with experimentally determined persistence length *l*_p_ = 11 nm and contour length, *L* = 70 nm (26). Because loop closure occurs in solution prior to surface binding, we set *s* = 1 for 3-dimensional equilibration.

## Data availability

All data needed to evaluate the conclusions in the paper are present in the paper and/or the Supplementary Materials. Additional data related to this paper may be requested from the corresponding authors (kinggm@missouri.edu or fbarrera@utk.edu).

## Supporting information

This article contains supporting information.

## Conflict of interest

The authors declare that they have no conflicts of interest with the contents of this article.
