# Supplementary material for: Polymerization mechanism of the Candida albicans virulence factor candidalysin
Source: J Biol Chem. 2024 May 13;300(6):107370. doi: 10.1016/j.jbc.2024.107370 (PMC11193009; doi:10.1016/j.jbc.2024.107370)
Supplement: Supplemental Figures S1–S3 [file mmc1.pdf]

Supplementary Information

Polymerization mechanism of the *Candida albicans* virulence factor candidalysin

Katherine G. Schaefer, Charles M. Russell, Robert J. Pyron, Elizabeth A. Conley, Francisco N. Barrera, and Gavin M. King

Contents

Supplemental Figure S1. CL induces a C-laurdan fluorescence spectra change

Supplemental Figure S2. AFM volume histograms and subunit conversion

Supplemental Figure S3. Concentration dependence of all features in AFM

Supplementary Figure S4. Non-subtracted GP values in different solutions

Supplementary Figure S5. pH dependence of CL assembly

Supplementary Figure S6. Effect of ionic strength on persistence length

Supplementary Figure S7. CL simulations determine the effect of morphology on contour length

Supplementary Figure S8. Reducing salt changes particle trends

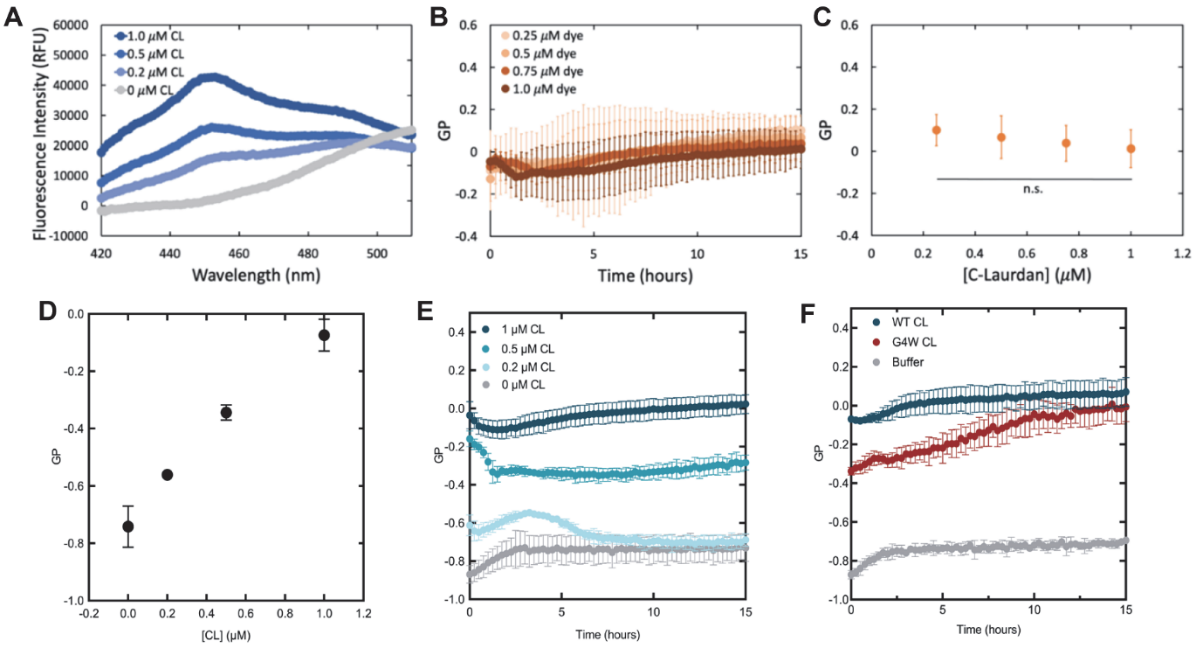

Figure S1. CL induces a C-laurdan fluorescence spectra change. (A) C-laurdan fluorescence spectra emission in the presence of CL suspended in phosphate buffered saline. **(B)** GP-values from different concentrations of C-laurdan in the presence of 0.5 μM CL. **(C)** No significant difference in GP amongst the C-laurdan concentrations in (B) after 15 hours. Error bars are representative of three replicates and statistics were performed using a Student's *t* test. (**D**) Non-subtracted GP-values corresponding to panel 1C. (**E-F**) show the raw GP values used to calculate ΔGP in panels 1E and 1F, respectively. Error bars represent the standard deviation of three independent experiments.

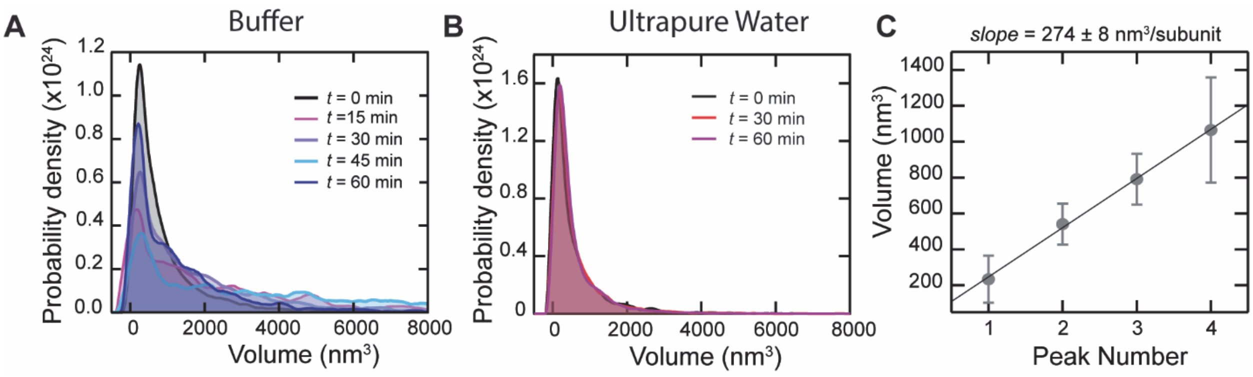

Figure S2. AFM volume histograms and subunit conversion. Histograms of AFM volumes over time are displayed for CL incubated in buffer (**A**) and ultrapure water (**B**). (**C**) Following the fitting of the buffer histogram at *t*=0 min with several Gaussians, the first four peak positions in (A) are fitted with a line in order to convert from volume to number of subunits.

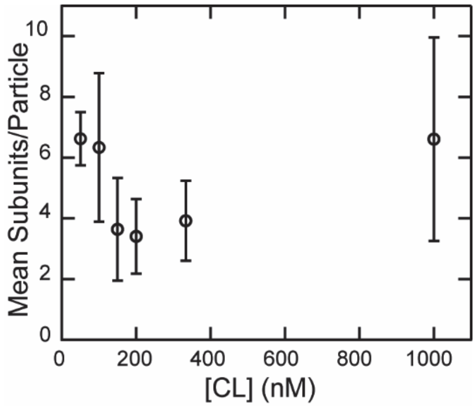

Figure S3. Concentration dependence of all features in AFM. The mean subunits/particle is plotted versus [CL] for all analyzed features.

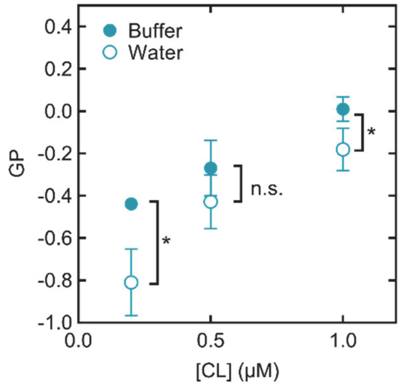

Figure S4. Non-subtracted GP values in different solutions. GP values of different CL concentrations after 4 hours in buffer or ultrapure water. Error bars are representative of three replicates and statistics were performed using a Student's *t* test.

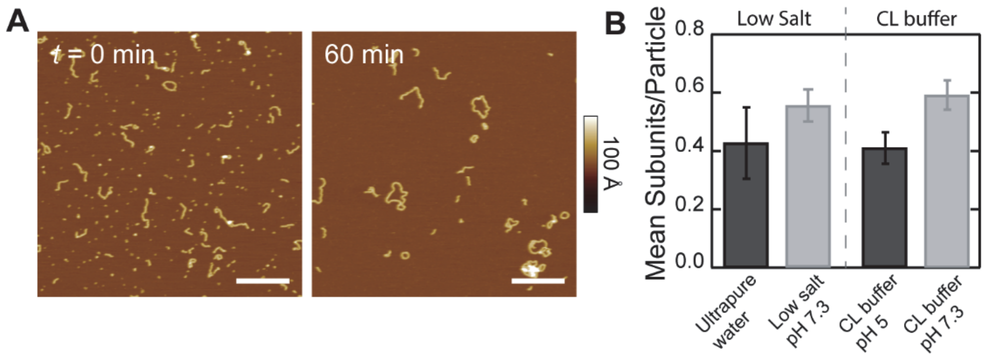

Figure S5. pH dependence of CL assembly. (**A**) AFM images show behavior of CL particles at 0 and 60 minute incubation times in acidic buffer (pH 5); scale bars = 200 nm. (**B**) The polymer seed (average subunits/particle for particles of octamer size or less) shows a decrease in size for low pH conditions (dark gray) regardless of salinity. At physiological pH (light gray), the average increases.

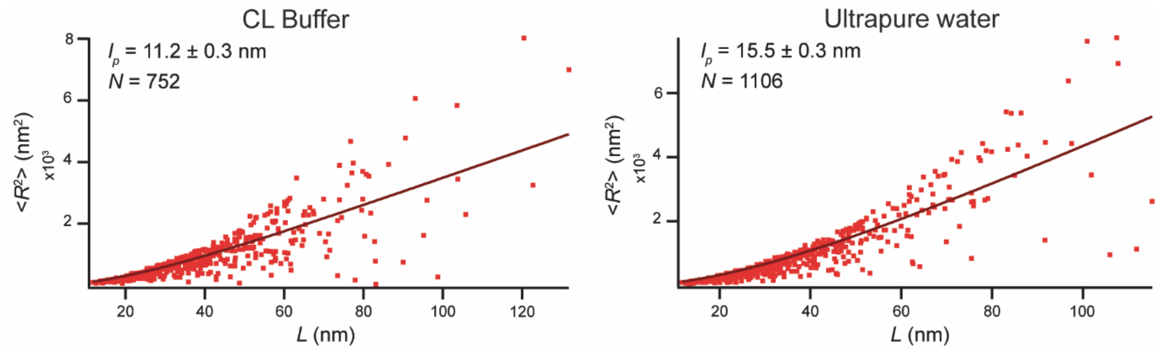

Figure S6. Effect of ionic strength on persistence length. The persistence length of CL polymers was quantified using AFM image data in conjunction with a custom algorithm (see Methods) which calculates the value using the Worm-like Chain Model. The values were determined from fits (solid lines) to the mean square end-to-end distances ($<R^2>$) versus the contour length (L) of $N \geq 752$ polymers. Salinity reduces the bending stiffness of the polymers, as evidenced by the decrease in persistence length, l_p, observed from ultrapure water (right) to buffer (left).

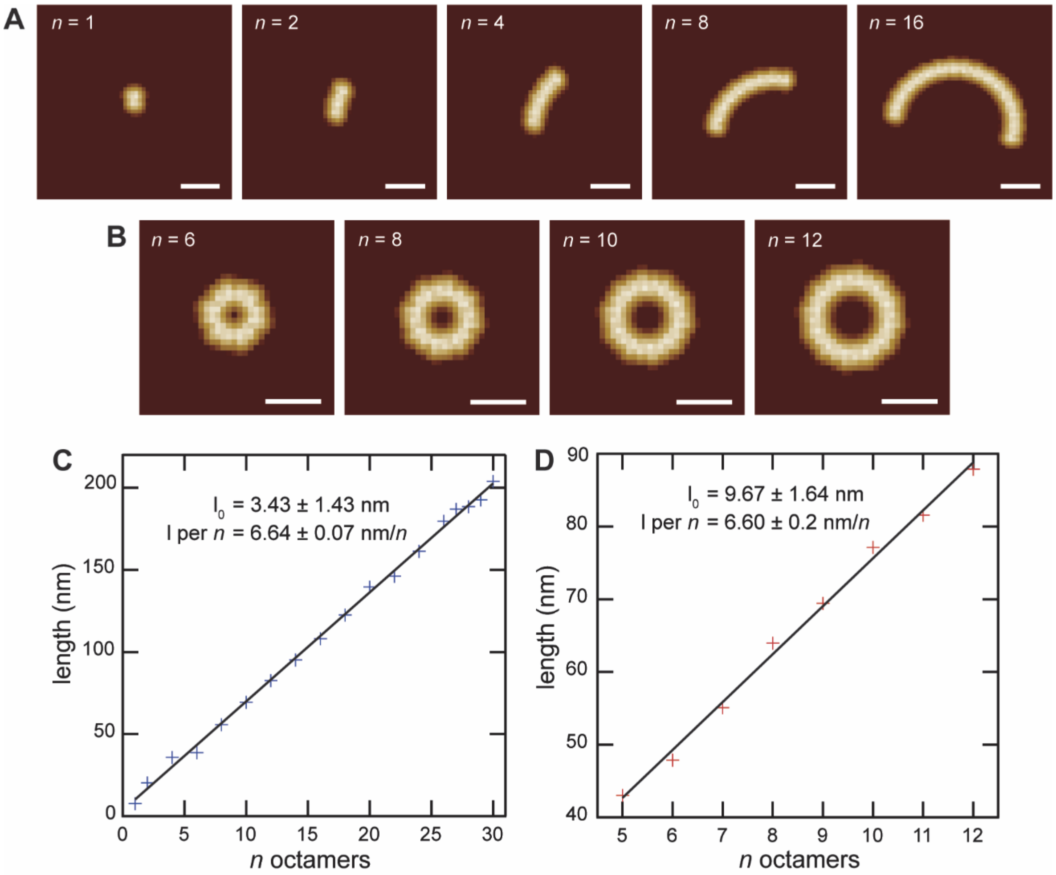

Figure S7. CL simulations determine the effect of morphology on contour length. Simulations were performed in order to understand the effect of tip convolution in the case of (**A**) a linear polymer and (**B**) loops; scale bars = 20 nm. Particles with increasing number of octamers (*n*) were simulated and the contour length of each plotted versus *n*. For linear and complex particles, we utilized the linear polymer fit in (**C**) to convert from contour length to number of subunits per particle. For loops, we used the fit for loops (**D**). In the case of individual subunits, the simple length of the simulated subunit was used, $l = 7.7$ nm.

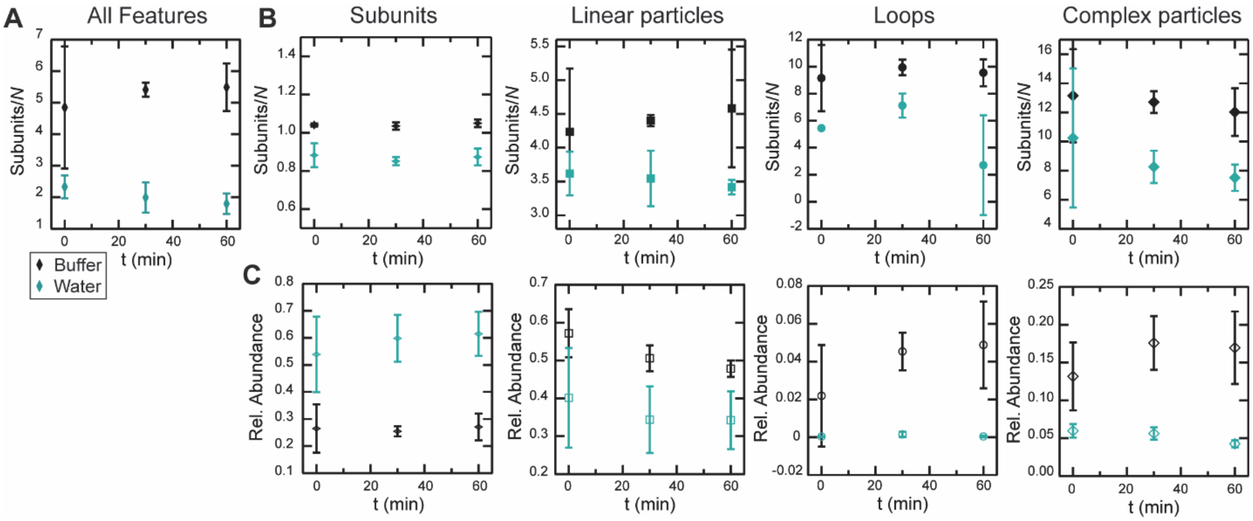

Figure S8. Reducing salt changes particle trends. AFM particle sorting is repeated for CL incubated in ultrapure water (*N* = 19,541). From the contour lengths, the mean Subunits/*N* is calculated for (**A**) all features and (**B**) other particle types. Overall, incubation in buffer (black) produces larger particles than incubation in ultrapure water (teal). (**C**) The relative abundance of subunits and linear particles dominates the ultrapure water samples. Very few loops and complex particles are observed in ultrapure water.
